# Hyperspectral imaging reveals early drought stress and associated molecular responses in lettuce for space agriculture

**DOI:** 10.1016/j.plaphe.2026.100223

**Published:** 2026-05-21

**Authors:** Lalit M. Kandpal, Ellen Garcia, Blake Costine, Chansong Hwang, Anirudha Dixit, LaShelle Spencer, Tie Liu, Aubrie O'Rourke, Insuck Baek, Moon Kim

**Affiliations:** a Environmental Microbial and Food Safety Laboratory, Agricultural Research Service, United States, Department of Agriculture, Beltsville, MD, 20705, USA; b Department of Horticultural Sciences, University of Florida, Gainesville, FL, 32611, USA; c Aetos Systems, Kennedy Space Center, FL, USA; d Department of Mechanical Engineering, University of Maryland, Baltimore County, 1000 Hilltop Circle, Baltimore, MD, 21250, USA; e Noetic Strategies, Kennedy Space Center, FL, USA; f Exploration Research and Technology, NASA Kennedy Space Center, Merritt Island, FL, USA

**Keywords:** Space agriculture, Drought stress, Hyperspectral imaging, Phenomics, Multi-omics integration, Early stress detection

## Abstract

In NASA's controlled-environment plant growth systems, early and autonomous detection of crop stress is critical for sustaining food production during long-duration space missions. Hyperspectral imaging (HSI) has proven effective for early stress detection, yet the molecular processes underlying diagnostically informative spectral signals remain poorly defined. Here, we present a two-stage phenomics-to-molecular framework to evaluate whether hyperspectral signatures associated with early drought detection correspond to coordinated molecular stress responses in lettuce. In the first stage, reflectance and fluorescence HSI were used to identify early drought detection windows in ‘Dragoon’ lettuce subjected to controlled water limitation over a 15-day treatment period with daily imaging. Classification models integrating reflectance and fluorescence outperformed single-modality models and achieved high accuracy as early as day after treatment (DAT) 4, reaching up to 97% at DAT 5. Partial least squares discriminant analysis (PLS-DA) identified predictive wavelengths concentrated in blue–green, red, and red-edge regions associated with chlorophyll absorption and photosystem II activity. In the second stage, independent transcriptomic and untargeted metabolomic profiles were integrated with hyperspectral signatures using MOFA2 to establish biological context. This analysis revealed a dominant drought axis characterized by early activation of ABA signaling, osmotic adjustment, phenylpropanoid metabolism, and lipid and membrane remodeling, with maximal molecular divergence at DAT 5, coinciding with peak hyperspectral classification performance. Notably, wavelengths optimized for early stress discrimination were systematically shifted toward shorter, optically efficient regions relative to those most strongly associated with downstream metabolic abundance, indicating that HSI primarily captures early structural and energetic consequences of molecular stress responses rather than direct biochemical composition. Together, these results demonstrate that hyperspectral imaging can function as a non-destructive, biologically interpretable molecular proxy for drought stress, providing a foundation for compact, hands-free sensing systems capable of distinguishing stress-specific plant states in space agriculture.

## 1 Introduction

In spacecraft and future Moon or Mars surface operations, advanced and autonomous monitoring systems will be essential for tracking plant health throughout the growth cycle. Such systems are critical not only for maintaining crop productivity and nutritional quality, but also for safeguarding astronaut health during long-duration missions. Among the environmental constraints encountered in controlled space-based plant production systems, water limitation represents one of the most significant stressors affecting plant growth and yield [1]. Insufficient water availability disrupts key physiological processes including photosynthesis, nutrient uptake, and transpiration, ultimately leading to reduced growth, yield penalties, and in severe cases, crop failure [[2], [3], [4]]. Early diagnosis of water stress is therefore essential to mitigate damage and enable timely intervention in controlled-environment agriculture systems [5], particularly for fresh, ready-to-eat crops such as leafy greens that are currently central to space food systems.

Existing approaches for monitoring plant water status in NASA growth chambers largely rely on traditional methods, including visual inspection, relative water content measurements, soil moisture sensing, leaf water potential measurements, or analysis of daily RGB images [6]. While informative, these techniques are often labor-intensive, episodic, or insufficiently sensitive to detect early physiological perturbations, limiting their utility for real-time decision-making in spaceflight environments. Consequently, there is a growing need for non-destructive, rapid, and automated sensing technologies capable of continuously assessing plant health in controlled space agriculture systems.

At the molecular level, analytical techniques such as nuclear magnetic resonance (NMR) and mass spectrometry (MS) have been widely used to characterize plant responses to abiotic and biotic stresses with high sensitivity and specificity [7,8]. However, the extensive sample preparation, destructive sampling, and reliance on sophisticated laboratory infrastructure make these approaches impractical for routine deployment in space. In response, substantial research efforts have focused on developing non-invasive alternatives, including thermal imaging and visible/near-infrared (VNIR) spectroscopy, which have demonstrated utility for detecting plant water stress across multiple crop species [[9], [10], [11], [12], [13], [14]].

Among these technologies, hyperspectral imaging (HSI) has emerged as a particularly powerful phenotyping tool in precision agriculture and plant phenomics [1]. Unlike conventional RGB imaging, which captures information in three broad spectral bands, or point-based spectroscopic approaches that lack spatial context, HSI acquires hundreds of contiguous spectral bands across the electromagnetic spectrum [[15], [16], [17]]. This enables detection of subtle, spatially resolved physiological changes that precede visible symptoms of stress. Accordingly, HSI has been successfully applied to detect abiotic stress responses in diverse crop systems, including raspberry [18], barley [19], and tomato [20]. Recent work in Plant Phenomics has demonstrated that hyperspectral imaging, combined with machine learning and multi-sensor phenotyping, enables robust stress detection and trait discrimination, and has started integrating transcriptomic and metabolomic data to provide biological context for observed phenotypes [[21], [22], [23]]. However, whether hyperspectral signatures reflect dynamic, time-sensitive molecular regulation and metabolic state transitions, rather than serving solely as correlates of stress phenotype, remains largely unresolved.

While HSI excels at early stress detection, interpreting the biological meaning of diagnostically informative spectral signatures remains a key challenge. Multi-omics approaches, including transcriptomics and metabolomics, provide a complementary framework for resolving the molecular processes underlying drought responses, particularly across pathways associated with abscisic acid (ABA) signaling [24], osmotic adjustment [25], secondary metabolism [26], and membrane remodeling [27]. Mechanistic links between spectral variation and molecular processes have started to emerge. Chlorophyll and carotenoid pigments dominate reflectance features in the blue (450–500 nm), red (640–680 nm), and red-edge (700–740 nm) regions [28,29], while mesophyll structure and leaf turgor influence near-infrared scattering between 750 and 1300 nm [30]. Leaf water content produces diagnostic absorption features near ∼970, 1200, 1450, and 1940 nm [31], and C–H functional groups in lipids and cuticular waxes contribute to short-wave infrared variation across 1700–2500 nm [32,33]. Establishing these mechanistic connections is especially important for space agriculture, where compact, autonomous sensing systems must infer plant physiological status without destructive sampling.

In this study, we investigate whether hyperspectral imaging can serve as a biologically interpretable proxy for underlying molecular drought responses in a space-relevant leafy crop grown under terrestrial controlled-environment conditions using a two-stage analytical framework. Building on our prior work that established a hyperspectral imaging platform [6] and identified early drought detection windows in controlled-environment lettuce production [34], the present study advances beyond stress classification to resolve the biological meaning of diagnostically informative spectral signatures through integrated transcriptomic and metabolomic analysis. In the first stage, hyperspectral imaging was used to identify early drought detection windows and diagnostically informative spectral features in ‘Dragoon’ lettuce (*Lactuca sativa* L.) subjected to controlled water limitation. In the second stage, independent transcriptomic and metabolomic profiling was integrated with hyperspectral signatures to establish biological context for the observed phenotypic signals. By explicitly linking predictive spectral regions to coordinated gene expression and metabolic responses, this work advances hyperspectral phenotyping beyond stress classification toward mechanistic interpretation, and provides a foundation for developing transferable, hands-free phenotyping strategies capable of distinguishing stress-specific plant states across environmental stressors, cultivars, and species in controlled and spaceflight agricultural systems.

Unlike conventional crop phenomics, where spectral features are used primarily for trait characterization, or stress classification, the framework presented here establishes a direct linkage between hyperspectral signature and underlying molecular processes. By integrating phenomic and multi-omics data, hyperspectral features are interpreted as ‘molecular proxies’ of coordinated physiological states, enabling biologically grounded, non-destructive inference of plant stress.

## 2 Materials and methods

### 2.1 Plant material, experimental design, and growth conditions

**Fig. 1:**
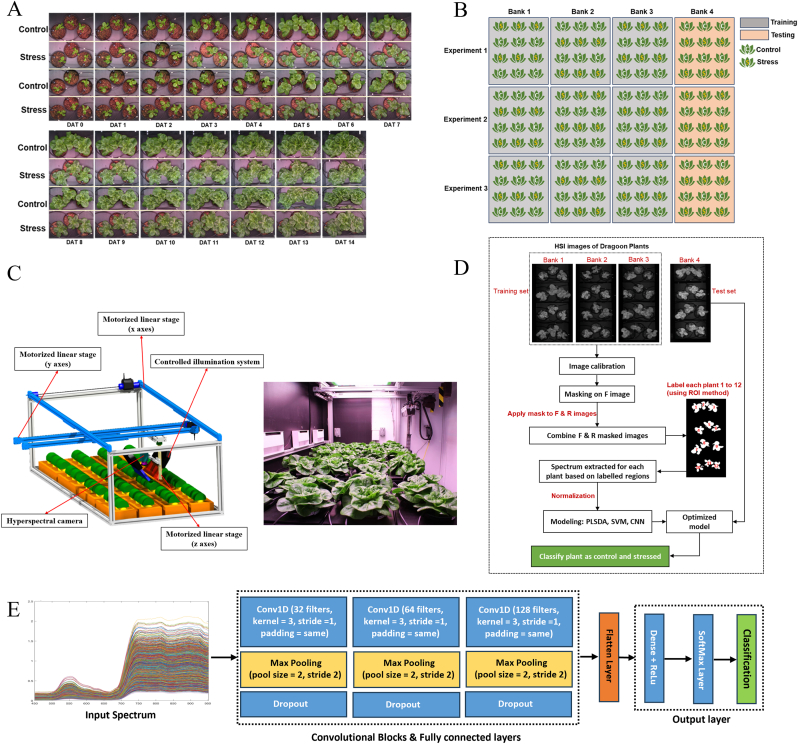
Experimental design, hyperspectral imaging system, and analysis workflow for drought stress detection in ‘Dragoon’ lettuce. (A) Time-series RGB images of representative plants from DAT 0 to DAT 14 under control and drought conditions in Experiment 1; the drip irrigation line used for automated water management is visible. (B) Experimental layout across three growth experiments showing plant distribution within banks and sample allocation for model training, testing, and independent validation. (C) Automated gantry-style hyperspectral imaging platform installed in a controlled-environment chamber at NASA Kennedy Space Center, integrating XYZ motion control, hyperspectral camera, and programmable illumination for reflectance and fluorescence imaging. (D) Hyperspectral data processing and classification workflow, including image calibration, plant masking, spectral extraction, preprocessing, model training, and evaluation using reflectance (R), fluorescence (F), and combined datasets. (E) One-dimensional convolutional neural network (1D-CNN) architecture used for hyperspectral reflectance-based drought stress classification.

‘Dragoon’ lettuce (*Lactuca sativa* L.) was selected as a space-relevant model crop to investigate drought stress responses using hyperspectral imaging (Fig. 1A). This cultivar has been identified by NASA as a promising candidate for space missions due to its high Crop Readiness Level (CRL) [35]. The study was comprised of six controlled-environment growth experiments conducted at NASA Kennedy Space Center (KSC, FL, USA; sea level, ∼101 kPa). Experiments 1–3 in the present study correspond to Experiments 2–4 reported in Kim et al. (2025) and were used here for hyperspectral model development, with experiment numbering adjusted to reflect the inclusion of additional downstream omics-focused growouts. Experiments 4–6 (May 8–22, 2024; June 26–July 10, 2024; August 14–28, 2024) were used exclusively for transcriptomic and metabolomic analyses and HSI from these datasets were further tested in the classification model to ensure accurate prediction (Table 1). In all experiments, plants were imaged from day after treatment (DAT) 0 to DAT 14, corresponding to 14–28 days after planting (DAP).

**Table 1:** Summary of experimental design, including dates, number of banks, and sample distribution per experiment.

Experiment #	Imaging period	# of Banks	Samples/bank	Datasets/DAT	Total datasets (15 Days)
1	June 01–15, 2023	4	12 (6 control + 6 stressed)	48	720
2	July 11–25, 2023	4	12 (6 control + 6 stressed)	48	720
3	Jan 25 – Feb 08, 2024	4	12 (6 control + 6 stressed)	48	720
4	May 8-22, 2024	4	12 (6 control + 6 stressed)	48	720
5	June 26- July 10, 2024	4	12 (6 control + 6 stressed)	48	720
6	August 14-28, 2024	4	12 (6 control + 6 stressed)	48	720

Plants were grown in a controlled-environment chamber (Percival PGW-48, IA) under constant conditions relevant to crewed space exploration: air temperature 23 °C, relative humidity 50%, and CO_2_ concentration 3000 ppm. Illumination was provided by Icarus Li2 LED fixtures (Bio Lighting, Melbourne, FL) delivering a photosynthetic photon flux density (PPFD) of 300 μmol m^−2^ s^−1^ using broad-spectrum white light (400–700 nm). Growth media consisted of a 70:30 (v/v) mixture of autoclaved professional growing mix and arcillite (Turface Profile Elite, PROFILE Products LLC, IL), with a 2 cm arcillite drainage layer at the base of each pot. Pots were weighed to ensure consistent media volumes across treatments. Each experiment consisted of four banks, with each bank containing twelve plants (six control and six drought-stressed), yielding 48 plants per DAT and 720 hyperspectral datasets per experiment. Control and drought-stressed trays were spatially alternated within the chamber to minimize positional effects (Fig. 1B). Volumetric water content (VWC) was monitored in real time using EC-5 soil moisture sensors (Meter Group, WA) connected to an Opto 22 industrial controller (Temecula, CA). All plants were maintained at approximately 45% VWC until drought treatment initiation at DAP 14 (DAT 0). Drought stress was imposed by reducing target VWMC to 20%, which was reached within four days (DAT 5), while control plants remained at ∼45% VWC. Irrigation was automatically triggered based on sensor feedback to maintain treatment-specific VWC setpoints.

### 2.2 Hyperspectral imaging system, data acquisition, and modeling

Hyperspectral imaging was performed using an automated gantry-style imaging system integrated within a controlled-environment growth chamber at NASA Kennedy Space Center (KSC, FL, USA), as previously described (Qin et al., 2022; Kim et al., 2025) (Fig. 1C). The system consists of a motorized XYZ translation stage and a line-scan hyperspectral sensing unit optimized for non-destructive plant phenotyping under space-relevant growth conditions. Hyperspectral data were acquired using a line-scan VNIR hyperspectral camera (MV.C VNIR; Headwall Photonics, Bolton, MA, USA) equipped with a block Offner spectrograph and a 12-bit CMOS detector (1936 × 1216 pixels), capturing 342 contiguous spectral bands spanning 400–1000 nm with 1024 spatial pixels per line. A 5 mm focal length wide-angle lens (Edmund Optics, Barrington, NJ, USA) was used to image approximately 40 cm across each plant row. A Wratten 2A gelatin filter (>400 nm; Kodak, Rochester, NY, USA) was installed to suppress residual UV-A illumination and second-order spectral interference near 730 nm.

Illumination was provided by programmable LED line lights (Metaphase Technologies, Bristol, PA, USA) delivering discrete reflectance wavelengths at 428, 650, 810, 850, 890, 910, and 915 nm, and ultraviolet-A excitation at 365 nm for fluorescence imaging. Light intensities were independently controlled using programmable six-channel dimming controllers. A calibrated reflectance reference panel (50 × 5 cm; Labsphere, North Sutton, NH, USA) was positioned within the field of view for flat-field correction. Hyperspectral data were collected daily from day after treatment (DAT) 0 to DAT 14 for each experiment. Each experiment consisted of four banks, with each bank containing 12 plants (six control and six drought-stressed). Imaging was performed in both reflectance and fluorescence modes, yielding 48 hyperspectral datasets per DAT per experiment. Radiometric calibration of reflectance images was performed using white and dark reference images collected immediately following each scan. Fluorescence images were corrected using dark reference subtraction only. Reflectance calibration was performed according to Equation:

$$I_{R\left(calibrated\right)}=\frac{I_{R}-I_{D}}{I_{W}-I_{D}}\times 100$$

where $I_{R\left(calibrated\right)}$ is the corrected image, $I_{R}$ is the raw sample image, $I_{D}$ is the dark reference image, and $I_{W}$ is the white reference image.

Following calibration, plant pixels were segmented from background using fluorescence-based thresholding, followed by morphological refinement. Binary masks were applied to both fluorescence and reflectance images to extract plant-only spectra. Individual plants were labeled using polygon-based regions of interest, and pixel-level spectra were randomly extracted for downstream modeling (Fig. 1D). Spectral data were preprocessed using several commonly used preprocessing methods, including normalization, Savitzky-Golay, multiplicative scatter correction (MSC) and standard normal variate (SNV). After comparing their effects on classification performance, range normalization followed by moving-mean smoothing was selected, as it consistently yielded the highest accuracy.

Supervised machine learning models were developed to classify control and drought-stressed plants using partial least squares discriminant analysis (PLS-DA) [36], support vector machines (SVM) [37], and one-dimensional convolutional neural networks (1D-CNN) [38,39]. Models were trained using reflectance-only, fluorescence-only, and combined reflectance–fluorescence datasets. Data from Experiments 1–3 were randomly split into training (70%) and test (30%) sets using Banks 1–3, with five-fold cross-validation applied during training. To evaluate model performance, samples from Bank 4 were completely withheld and used solely for independent validation (Fig. 1B), ensuring no overlap at either the plant or pixel level. Furthermore, models trained on data from Experiments 1–3 were tested on Experiments 4–6 as an external validation set, comprising newly grown and imaged plants under similar conditions, to assess their ability to accurately distinguish between drought and control classes.

The total number of pixel spectra used for model development and testing at each DAT is summarized in Table 2. In PLS-DA models, beta coefficients were extracted to identify wavelengths contributing most strongly to drought discrimination; beta coefficients represent the weighted contribution of each wavelength to the discriminant function. A SVM classifier with a Radial Basis Function (RBF) kernel was implemented for classification. The regularization parameter (C) was set to 1, while the kernel scale (γ) was automatically estimated from the data using 5-fold cross-validation to ensure robustness and reduce the risk of overfitting. The CNN network consisted of three convolutional layers with 32, 64, and 128 filters respectively, each using a kernel size of 3, stride 2, and same padding. Each convolution layer is followed by a ReLu activation function. Max pooling layers with pool size 2 and stride 2 were included after each convolutional layer to reduce the feature dimensionality while retaining the most informative spectral features. The convolutional blocks are followed by a flatten layer and a fully connected layer with 512 neurons, ReLU activation, and dropout rate of 0.5 to reduce overfitting. The final classification layer consists of two neurons with a softmax activation function. The network was trained using Adam optimizer with initial rate of 1 × 10^−3^, a mini-batch size of 32 for 30 epochs. Cross-entropy loss was used as the objective function.

**Table 2:** Total number of selected pixel data utilized in the PLS-DA, SVM, and CNN models for classification of control and stressed plants.

Day		Train set	Test set
DAP	DAT		
14	0	2803	640
15	1	3088	1005
16	2	3993	1350
17	3	6495	2004
18	4	8982	2806
19	5	12347	3726
20	6	16280	4868
21	7	20649	6111
22	8	25168	7540
23	9	29011	8703
24	10	33430	9720
25	11	35849	10910
26	12	39496	11648
27	13	42396	12699
28	14	44750	15016

### 2.3 Transcriptomic profiling and analysis

Transcriptomic analyses were conducted using three independent growth experiments (Experiments 4–6) grown under identical environmental conditions to the three hyperspectral model development experiments. Leaf tissue was collected from control and drought-stressed plants at DAT 0, DAT 5, and DAT 14. For each timepoint, tissue from nine individual plants per treatment was flash-frozen in liquid nitrogen and stored at −80 °C until processing at NASA Kennedy Space Center (KSC, FL, USA). Frozen tissue was ground to a fine powder using a cryogenic mill, and total RNA was extracted from 200 mg of tissue using TRIzol reagent (Invitrogen, USA), followed by cleanup with the PureLink RNA Mini Kit (Invitrogen, USA). RNA concentration was quantified using the Qubit™ RNA High Sensitivity assay (Invitrogen, USA), and integrity was assessed using an Agilent 2100 Bioanalyzer (Agilent Technologies, USA). High-quality RNA was submitted to Azenta Life Sciences for library preparation and sequencing. Libraries were prepared using Illumina TruSeq mRNA library preparation kits with TruSeq UD Indexes V2 dual indices according to manufacturer protocols and sequenced on an Illumina NovaSeq X Plus platform using paired-end 2 × 150 bp reads, generating approximately 2 billion reads.

Raw RNA-seq reads were processed following Overbey et al. [40]. Adapter trimming and quality filtering were performed using TrimGalore (v0.6.10) with parameters: --paired, --quality 30, --stranded_illumina, and --cores 4. Reads shorter than 20 bp were discarded, and only read pairs in which both mates passed filtering were retained. Trimmed reads were aligned to the *Lactuca sativa* reference genome (Lsat_Salinas_v11; GCF_002870075.4) using STAR [41]. Gene-level counts were generated using RSEM (Li and Dewey, 2011).

Differential expression analysis was conducted in R using edgeR [42]. Genes with |log_2_FC| ≥ 1 and p <0.05 were considered differentially expressed. K-means clustering of DEGs was performed using logCPM values, with the optimal number of clusters determined using the Silhouette method [43]. Heatmaps were generated using the R package *heatmap*. Gene Ontology enrichment of DEG clusters was performed using the enricher function in clusterProfiler (v4.8.2; Wu et al., 2021), with adjusted p-values <0.05 and a minimum of two genes per term. KEGG pathway enrichment was conducted using enrichKEGG with annotations retrieved via biomaRt (Durinck et al., 2009), applying an adjusted p-value threshold of 0.05.

### 2.4 Metabolomic profiling and analysis

Untargeted metabolomic analyses were performed on the corresponding plants from the independent growth experiments (Experiments 4–6) also used for transcriptomic profiling. Using the leaf tissue from control and drought-stressed plants that was harvested at DAT 0, DAT 5, and DAT 14, flash-frozen in liquid nitrogen, lyophilized, and ground to a fine powder, samples were shipped on dry ice to Metabolon, Inc. (Morrisville, NC, USA) for analysis using their global discovery metabolomics platform. Metabolite profiling was conducted using four ultra-performance liquid chromatography–tandem mass spectrometry (UPLC–MS/MS) methods optimized for hydrophilic acidic positive ions, hydrophobic acidic positive ions, basic negative ions, and HILIC-eluted negative ions. Raw metabolite abundances were processed following standard Metabolon workflows, including median normalization and log transformation.

Metabolomics data were analyzed separately for DAT 0, DAT 5, and DAT 14 to identify timepoint-specific drought responses. Partial least squares discriminant analysis (PLS-DA) was performed independently for DAT 5 and DAT 14 contrasts between drought-stressed and control plants using the CIMCB metabolomics workflow (Centre for Integrative Metabolomics and Computational Biology; https://github.com/CIMCB/cimcb) implemented in a Python/Jupyter environment. Variable Importance in Projection (VIP) scores were extracted from each model, and metabolites with VIP >1 were considered significant contributors to drought discrimination. Overlapping and timepoint-specific drought-responsive metabolites were identified using Venny 2.1.0. Integrated pathway analysis and visualization were performed using PaintOmics 4 [44] , mapping transcriptomic and metabolomic data onto curated biological pathways to facilitate functional interpretation of multi-omics responses.

### 2.5 Multi-omics and hyperspectral imaging integration using MOFA2

To integrate molecular drought responses with hyperspectral phenotypes, multi-omics factor analysis was performed using MOFA2 (v1.7.3) in R (v4.3.2). Transcriptomic and metabolomic datasets from DAT 0, DAT 5, and DAT 14 were used as primary molecular views. RNA-seq data were filtered to retain genes with CPM >1 in at least three samples, transformed to logCPM using edgeR, and z-score normalized. Metabolite abundances were log10-transformed and z-score normalized. All molecular matrices were aligned by sample ID and timepoint prior to integration. Hyperspectral imaging was incorporated at the phenotype level by linking molecular latent factors to hyperspectral drought signatures derived from the independent classification models. Specifically, hyperspectral drought status and timepoint-resolved classification performance were used to biologically anchor latent molecular factors to phenotypic drought progression rather than to individual spectral bands or pixels.

MOFA2 models were trained using a Gaussian likelihood with eight latent factors and default evidence lower bound (ELBO) convergence criteria. Factor scores were evaluated using linear models of the form Factor ∼ treatment + DAT + treatment × DAT, with significance defined as p < 0.05. Feature loadings were extracted for each factor, and contributors were defined as features within the top 5% of absolute loading values. To characterize coordinated molecular responses underlying hyperspectral drought phenotypes, Pearson correlations between feature values and factor scores were computed using cor. test in R, applying thresholds of |r| ≥ 0.3 and FDR <0.05 (Benjamini–Hochberg). Strong gene–metabolite associations (|r| ≥ 0.7, FDR <0.05) were retained for network construction and visualization using igraph (v2.0.1). Latent factors significantly associated with drought treatment and timepoint were subsequently interpreted in the context of hyperspectral classification accuracy and diagnostically informative spectral regions to establish biological meaning for early drought detection signals.

## 3 Results

### 3.1 Hyperspectral reflectance and fluorescence exhibit time-dependent divergence between control and drought-stressed plants

**Fig. 2:**
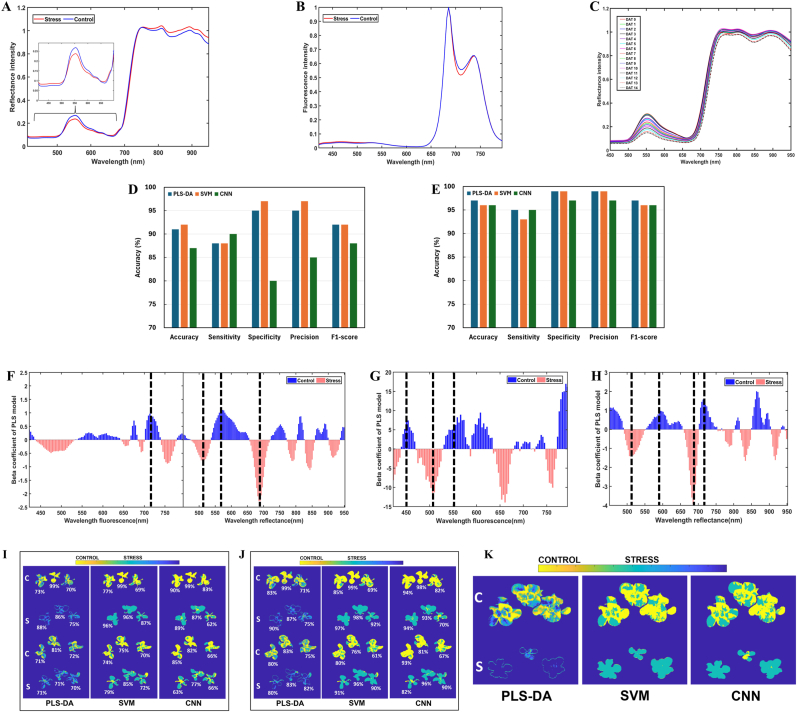
Hyperspectral spectral signatures, classification performance, and spatial drought detection in Dragoon lettuce. (A) Mean reflectance spectra of control and drought-stressed plants at DAT 4. (B) Mean fluorescence spectra of control and drought-stressed plants at DAT 4. (C) Temporal evolution of reflectance spectra in drought-stressed plants from DAT 0 to DAT 14. (D–E) Classification performance metrics (accuracy, sensitivity, specificity, precision, F1-score) for combined reflectance–fluorescence models at DAT 4 (D) and DAT 5 (E) using PLS-DA, SVM, and CNN. (F–H) PLS-DA beta coefficients at DAT 5 for combined (F), combines fluorescence and reflectance models, indicating wavelength contributions to class separation (blue: control; red: stress) (G) fluorescence-only (H), and reflectance-only. (I–J) Pixel-level classification maps for Experiment 1, Bank 4 samples at DAT 4 (I) and DAT 5 (J) using combined data, showing spatial discrimination of control (C) and stressed (S) plants with per-plant accuracies. (K) Classification maps for an independent growout (Experiment 4) generated using DAT 5–trained models, demonstrating generalization across experiments.

Fig. 2A and B shows the average reflectance and fluorescence spectra of control and drought-stressed Dragoon lettuce plants at DAT 4 after range normalization. In the reflectance spectra (Fig. 2A), control plants exhibited higher reflectance intensity in the green region (500–600 nm) compared to stressed plants, with additional differences observed in the blue (400–500 nm) and red-edge regions (600–700 nm) [45].

The fluorescence spectra (Fig. 2B) exhibited overall similar spectral profiles between control and stressed plants, with only minor differences detected across wavelengths. Slightly reduced fluorescence intensity was observed in stressed plants in the blue region (400–500 nm) relative to controls. Temporal changes in reflectance spectra of stressed plants from DAT 0 to DAT 14 are shown in Fig. 2C. In the green region (∼550 nm), reflectance intensity decreased progressively over time, with higher reflectance observed during early time points (DAT 0–4) followed by lower reflectance values at later stages (DAT 5–14), indicating a gradual shift in spectral response as drought stress progressed.

### 3.2 Hyperspectral classification accuracy increases sharply at DAT 4–5, defining an early drought detection window

Classification performance for PLS-DA, SVM, and CNN models using reflectance (R), fluorescence (F), and combined reflectance–fluorescence (R + F) datasets across DAT 0–14 is summarized in Fig. 2D and E and Table 3. During early time points (DAT 0–3), classification accuracy remained low across all models and input types. On DAT 0, the combined model achieved accuracies of 55% (PLS-DA), 45% (SVM), and 41% (CNN), with similar performance observed through DAT 3, indicating limited spectral separability between control and drought-stressed plants during this period.

**Table 3:** Performance results of classifying control and stressed samples using PLS-DA, SVM and CNN models on the test set.

DAT	Reflectance (R)			Fluorescence (F)			Combined (R + F)		
	PLS-DA (%)	SVM (%)	CNN (%)	PLS-DA (%)	SVM (%)	CNN (%)	PLS-DA (%)	SVM (%)	CNN (%)
0	47	52	47	34	53	41	55	45	41
1	63	63	50	54	61	55	68	68	55
2	66	64	50	55	57	55	66	66	54
3	77	78	72	69	71	58	79	82	64
4	86	89	87	81	83	72	91	92	87
5	96	94	96	96	96	91	97	96	96
6	96	95	97	97	96	93	96	97	94
7	96	96	94	96	96	92	97	97	94
8	95	95	90	95	97	90	96	97	91
9	91	92	83	93	95	83	94	95	89
10	88	91	82	93	95	83	94	95	88
11	88	89	83	91	95	91	93	94	91
12	86	88	80	92	94	90	92	93	89
13	86	90	83	92	92	84	89	93	89
14	86	88	81	84	84	80	86	88	89

A marked increase in performance was observed at DAT 4, particularly for models trained on the combined dataset. On DAT 4, combined-model accuracies reached 91% (PLS-DA), 92% (SVM), and 87% (CNN), whereas reflectance-only and fluorescence-only models did not exceed 83% accuracy. Corresponding performance metrics for the combined SVM model at DAT 4 included 88% sensitivity, 97% specificity, 97% precision, and an F1-score of 92% (Fig. 2D).

By DAT 5, classification performance exceeded 90% across all models and input types. The combined PLS-DA model achieved 97% accuracy, with 95% sensitivity, 99% specificity, 99% precision, and an F1-score of 97% (Fig. 2E), while SVM and CNN models trained on combined data each reached 96% accuracy with F1-scores of 96%. Reflectance-only and fluorescence-only models similarly achieved high accuracy from DAT 5 onward. From DAT 6 to DAT 10, all classifiers maintained stable performance, with accuracies ranging between approximately 90% and 97% across models and input types. At later time points (DAT 11–14), a modest decline in accuracy was observed for some models; however, all approaches continued to achieve high classification accuracy through DAT 14.

To identify spectral features contributing to drought classification, PLS-DA beta coefficients were examined at DAT 5 (Fig. 2F–H). The key contributing wavelengths were identified based on prominent peaks and trough (local maxima and mina) in the beta coefficient profiles and are indicated in the figure using vertical dashed lines. Positive coefficients associated with control plants were primarily observed in the green region (approximately 550–600 nm), whereas negative coefficients associated with drought-stressed plants were concentrated in the blue (450–500 nm) and red (650–700 nm) regions. Additional weighting was observed near 660–680 nm across models. These beta coefficient patterns were consistent across reflectance-only, fluorescence-only, and combined datasets, indicating reproducible wavelength contributions to class separation at the onset of detectable stress. The majority of the highlighted wavelengths correspond to spectral regions associated with plant pigments, primarily chlorophyll and carotenoids [13,46].

### 3.3 Early drought classification generalizes to independent growouts

To evaluate model generalizability, classification models trained on combined reflectance and fluorescence (R + F) data from Experiments 1–3 were applied to independent growouts (Experiments 4, 5, and 6), which were not used during model training and were also used for transcriptomic and metabolomic analyses. These experiments consisted of new sets of control and drought-stressed ‘Dragoon’ lettuce plants grown and imaged under identical environmental and hyperspectral acquisition conditions.

Pixel-level classification maps for Experiment 4 generated using models trained at DAT 4 and DAT 5 are shown in Fig. 2I and J. All three classifiers, PLS-DA, SVM, and CNN, successfully distinguished control (C) and drought-stressed (S) plants. Spatial predictions were consistent across classifiers and across individual plants, indicating robust discrimination of drought stress at the pixel level.

Classification results from Experiments 5 and 6 are shown in Fig. 2K. Classification patterns were consistent with those observed in Experiment 4, demonstrating that models trained during the early drought detection window generalized effectively across independent growouts. These results confirm that hyperspectral models trained on early drought time points retained predictive performance when applied to unseen biological replicates.

### 3.4 Global transcriptomic and metabolomic profiling reveals early activation of ABA, redox, and phenylpropanoid pathways with concurrent repression of photosynthetic and growth programs

**Fig. 3:**
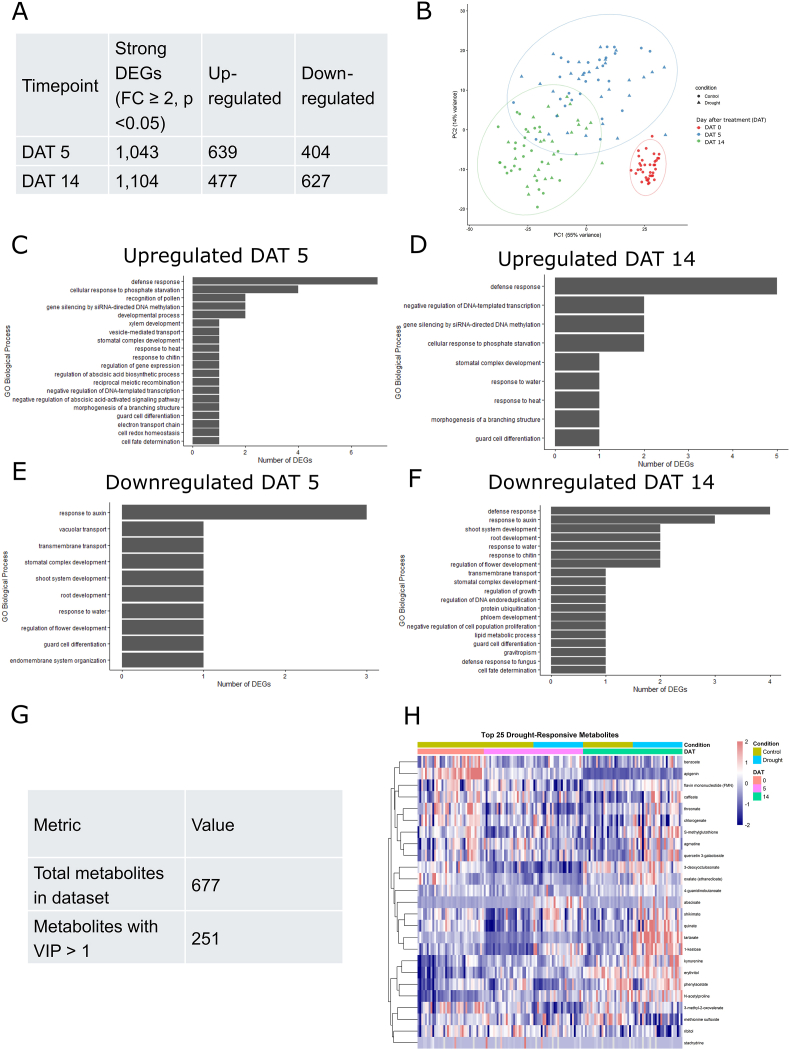
Global transcriptomic and metabolomic responses to drought at DAT 5 and DAT 14. (A) Numbers of strongly regulated genes (|log_2_FC| ≥ 1, p < 0.05). (B) PCA showing developmental structure with within-stage drought effects. (C–F) GO enrichment of up- and down-regulated genes highlighting activation of stress pathways and repression of growth-related processes. (G) Summary of metabolomics dataset and VIP>1 features. (H) Heatmap of the top 25 discriminant metabolites.

Drought triggered extensive transcriptional remodeling (Supplementary Data 1) at both DAT 5 and DAT 14 (Fig. 3A and B). At DAT 5, 15,452 genes were differentially expressed (p < 0.05), including 8151 upregulated and 7301 downregulated transcripts, of which 1043 met the strong-response threshold (|log_2_FC| ≥ 1). At DAT 14, 13,491 genes were significantly altered (7201 upregulated, 6290 downregulated), with 1104 genes exhibiting |log_2_FC| ≥ 1. These results indicate a robust and sustained transcriptional response to drought, with early stress marked by widespread induction of responsive genes and later stages characterized by an increasing proportion of repressed regulatory and metabolic processes.

Gene Ontology enrichment of strongly upregulated genes revealed consistent activation of abscisic acid (ABA)–responsive signaling, osmotic stress responses, reactive oxygen species detoxification, phenylpropanoid biosynthesis, lipid remodeling, and cell-wall modification pathways at both DAT 5 and DAT 14 (Fig. 3C and D). These functional categories reflect canonical drought-responsive programs that promote stress perception, cellular protection, and metabolic adjustment during water limitation.

In contrast, downregulated genes were enriched for photosynthesis, chloroplast organization, protein translation, nutrient assimilation, and cell-cycle–associated processes, indicating a coordinated shift away from growth and energy-intensive functions under sustained drought (Fig. 3E and F). Repression of photosynthesis- and chloroplast-associated gene sets was evident at both time points, consistent with early perturbation of chlorophyll-related processes and provide a molecular basis for the strong drought-discriminative hyperspectral signals observed in chlorophyll absorption and red-edge spectral regions.

Untargeted metabolomic profiling (Supplementary Data 2) identified 677 metabolites, of which 251 exhibited VIP >1 in PLS-DA contrasts between drought-stressed and control plants (Fig. 3G). The top 25 discriminant metabolites formed coherent biochemical clusters with distinct temporal dynamics (Fig. 3H). Amino acid–derived metabolites, including proline and N-acetylated amino acids, increased under drought at both DAT 5 and DAT 14, reflecting progressive osmotic adjustment. Organic acids associated with the shikimate and quinate pathways, along with phenylpropanoid intermediates such as feruloylquinate, also accumulated over time, indicating sustained activation of aromatic and secondary metabolism. In contrast, select lipid-associated metabolites, including sphingolipids and glycerophosphoglycerol species, displayed time-dependent responses, consistent with early membrane remodeling followed by later stabilization. Integrated pathway mapping using PaintOmics 4 [13]confirmed significant overlap between drought-responsive transcripts and metabolites within hormone signaling, phenylpropanoid biosynthesis, redox regulation, and lipid metabolism pathways (Supplementary Data 3), demonstrating coordinated multi-layer regulation of drought responses [47].

### 3.5 Identification and characterization of a dominant drought-associated latent factor linking hyperspectral, metabolomic, and transcriptomic variation

**Fig. 4:**
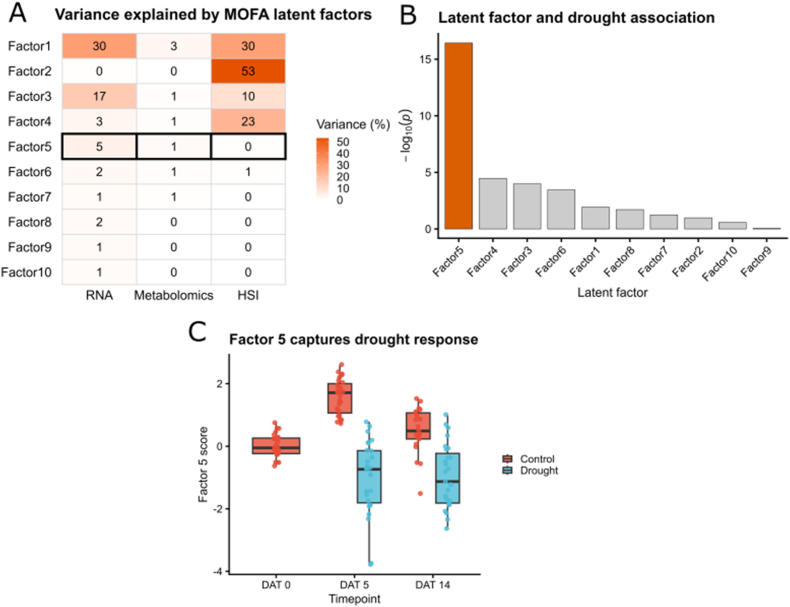
A dominant drought-associated latent factor links hyperspectral, metabolomic, and transcriptomic variation. (A) Variance explained by MOFA latent factors across transcriptomic, metabolomic, and hyperspectral (HSI) data layers. (B) Association of latent factors with drought treatment. (C) Distribution of Factor 5 scores across timepoints under control and drought conditions.

To integrate hyperspectral imaging (HSI) (Supplementary Data 4), metabolomic, and transcriptomic measurements collected from the same plants, a multi-omics factor analysis (MOFA2) model was fitted using 172 hyperspectral wavelengths, 677 metabolites, and genome-wide RNA-seq expression profiles across 129 shared samples (Supplementary Data 5). Examination of variance explained across all ten latent factors revealed that Factor5 captured the strongest shared biological signal across the three data layers (Fig. 4A). Analysis of variance (ANOVA) across latent factors further confirmed that Factor5 exhibited the most pronounced association with drought treatment among all factors (Fig. 4B).

Statistical testing of Factor5 scores using a linear model incorporating Treatment (drought vs. control), Timepoint, their interaction, and Experiment as a covariate demonstrated that Factor5 was the most strongly drought-associated latent axis (Treatment p = 2.43 × 10^−21^), with a significant effect of Timepoint (p = 3.72 × 10^−8^) and a significant Treatment × Timepoint interaction (p = 0.00148). No other latent factor exhibited a Treatment effect of comparable magnitude. Visualization of Factor5 scores across timepoints confirmed robust separation between drought-stressed and control plants (Fig. 4C). Control plants consistently exhibited positive Factor5 scores, whereas drought-treated plants exhibited negative scores, with maximal separation at the intermediate timepoint and partial convergence at the final timepoint, consistent with physiological acclimation rather than loss of drought-associated signal.

**Fig. 5:**
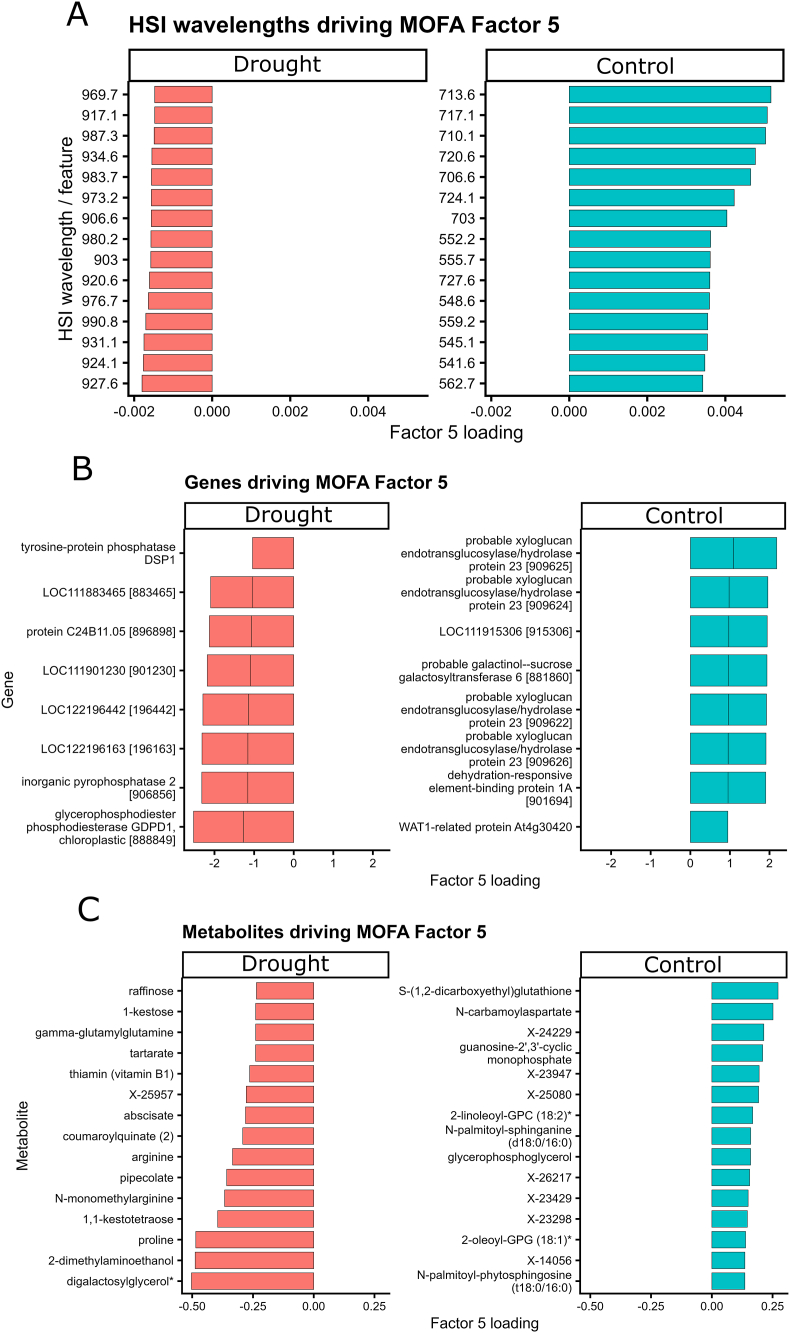
Spectral, transcriptomic, and metabolomic features contributing to the drought-associated latent factor. (A) Hyperspectral wavelength loadings for Factor 5. (B) Top gene loadings contributing to Factor 5. (C) Top metabolite loadings contributing to Factor 5.

Analysis of hyperspectral feature loadings revealed a structured and biologically interpretable spectral signature underlying Factor5 (Fig. 5A). Wavelengths with negative loadings, corresponding to drought-associated samples, were enriched in the near-infrared (NIR) region between approximately 900 and 1000 nm, a spectral region sensitive to leaf water content and internal leaf structure. In contrast, wavelengths with positive loadings, corresponding to control-associated samples, were concentrated in the red-edge region (∼700–730 nm) and the green visible region (∼530–570 nm), which are associated with chlorophyll content, pigment balance, and photosynthetic capacity. This opposing spectral structure indicates that Factor5 captures coordinated changes in hydration status, optical properties, and photosynthetic function rather than isolated wavelength effects.

Metabolomic feature loadings for Factor5 further supported its interpretation as a drought-response axis (Fig. 5B). The drought-associated side of the latent factor was driven by a coherent set of metabolites with established roles in drought stress physiology, including amino acid–derived osmolytes, lipid species associated with membrane remodeling, secondary metabolites, and hormone-related compounds such as abscisate, proline, and raffinose-related sugars [48]. Aggregation of Factor5-associated metabolites by chemical class showed that amino acid metabolism accounted for the largest number of significant associations (539), followed by lipids (408), secondary metabolism (240), carbohydrates (131), nucleotides (67), cofactors and prosthetic groups (40), and hormone metabolism (7). These results indicate that Factor5 reflects broad metabolic reprogramming encompassing osmotic adjustment, membrane composition, redox balance, and hormone-mediated stress signaling.

Transcriptomic loadings for Factor5 were concordant with these metabolic and spectral patterns (Fig. 5C). Genes contributing strongly to the latent factor included those involved in cell wall remodeling, carbohydrate metabolism, stress-responsive signaling, and chloroplast-associated processes. Genes associated with growth-related cell wall modification and photosynthetic function tended to load positively and were enriched in control plants, whereas genes linked to stress signaling, metabolic adjustment, and cellular protection tended to load negatively and were enriched in drought-stressed plants. The concordance between transcriptomic, metabolomic, and hyperspectral contributions supports interpretation of Factor5 as an integrated drought-response state rather than a layer-specific artifact.

**Fig. 6:**
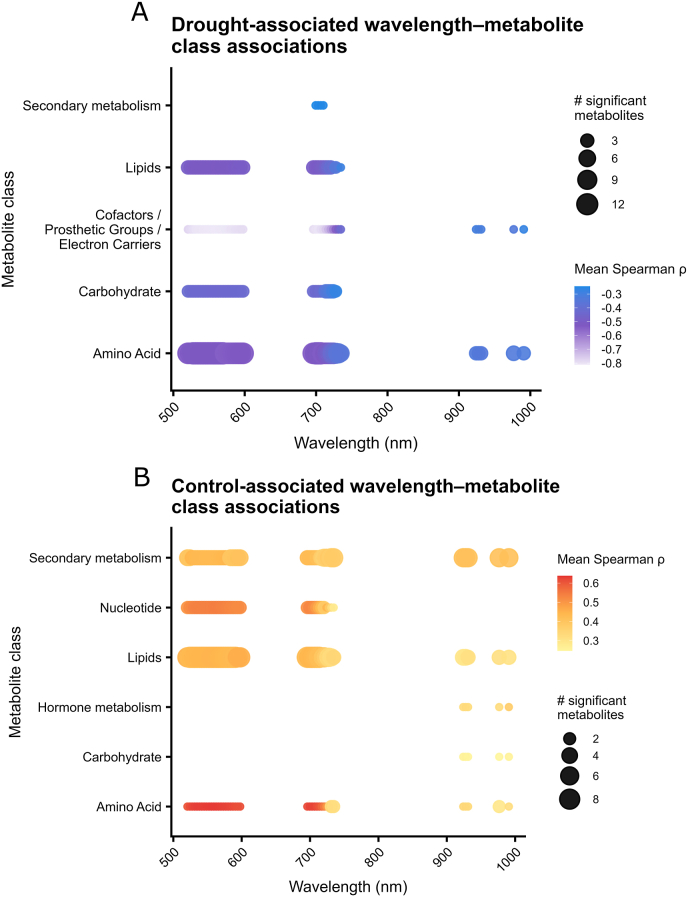
Wavelength–metabolite class associations under drought and control conditions. (A) Drought-associated wavelength–metabolite class correlations. (B) Control-associated wavelength–metabolite class correlations. Point size represents the number of significant metabolites, and color indicates mean Spearman correlation (ρ).

To directly link spectral variation to biochemical processes, a MOFA-guided wavelength–metabolite correlation analysis was performed using the 40 hyperspectral wavelengths and 80 metabolites with the highest absolute Factor 5 loadings, yielding 2280 pairwise tests (Supplementary Data 6). After Benjamini–Hochberg correction, 1432 associations remained significant (FDR <0.05). Significant associations were separated into drought-associated and control-associated subsets and summarized by metabolite class and spectral region. Drought-associated correlations were comprised of 622 significant associations (FDR <0.01), dominated by amino acid metabolism (n = 409), followed by lipids (n = 99), carbohydrates (n = 70), and cofactors/prosthetic groups/electron carriers (n = 40), with minimal representation from secondary metabolism (n = 4) (Fig. 6A). Correlation coefficients were consistently negative across drought-associated classes, with mean values ranging from −0.410 (carbohydrates) to −0.703 (cofactors/prosthetic groups/electron carriers). The strongest drought-associated metabolites included thiamin (vitamin B1), dimethylarginine (SDMA + ADMA), N-delta-acetylornithine, gamma-glutamylglutamine, and 2-palmitoyl-galactosylglycerol, along with additional amino acid–derived compounds such as oxindolylalanine, O-sulfo-tyrosine, arginine, histidine, and N-monomethylarginine. These associations spanned 521–991 nm and were most abundant in the green visible region (520–570 nm), with additional enrichment in the red-edge (680–740 nm) and NIR (>900 nm) regions.

Control-associated correlations comprised 554 significant associations (FDR <0.01), driven primarily by lipids (n = 280) and secondary metabolism (n = 155), with additional contributions from nucleotides (n = 66) and amino acids (n = 43) (Fig. 6B). In contrast to drought, correlation coefficients were consistently positive across all control-associated classes, with the highest mean values observed for amino acids (0.545) and nucleotides (0.509), followed by lipids (0.425) and secondary metabolism (0.412). The strongest control-associated metabolites included N-carbamoylaspartate, S-(1,2-dicarboxyethyl)glutathione, N-palmitoyl-sphinganine, N-palmitoyl-phytosphingosine, coumaroylquinate (5), and glycerophosphoglycerol, along with lipid species such as 2-linolenoyl-GPE (18:3)∗ and 2-oleoyl-GPE (18:1)∗ and additional metabolites including salidroside, guanosine-2′,3′-cyclic monophosphate, and 4-hydroxycinnamate. These associations also spanned 521–991 nm and were concentrated in the green visible region, with substantial representation in the red-edge and NIR regions. Importantly, negative correlations indicate inverse relationships between spectral intensity and metabolite signal (e.g., increases in one accompanied by decreases in the other), whereas positive correlations indicate coordinated variation (both increasing or decreasing together), highlighting fundamentally distinct patterns of spectral–metabolic coupling under drought versus control conditions.

Together, these results demonstrate that the hyperspectral regions driving drought discrimination correspond to coordinated molecular states involving transcriptional reprogramming and metabolic adjustment. While hyperspectral signals do not directly detect individual metabolites, they function as non-destructive proxies of integrated physiological responses, capturing coupled optical, biochemical, and transcriptional variation associated with drought stress.

## 4 Discussion

This study presents a comprehensive evaluation of hyperspectral imaging as a non-destructive tool for early detection of drought stress in *Lactuca sativa* ‘Dragoon’ grown under controlled-environment conditions. By integrating reflectance and fluorescence hyperspectral data with machine learning classification, we demonstrate that drought-induced physiological changes produce characteristic and time-dependent spectral signatures that can be detected prior to the appearance of visible stress symptoms. In particular, stress-related alterations were most pronounced in spectral regions associated with chlorophyll content and photosynthetic function, consistent with the known sensitivity of vegetation reflectance to early physiological perturbations under water limitation. The progressive decrease in reflectance in the green region (500–600 nm) under drought is consistent with transcriptomic evidence of downregulation of photosynthesis and chloroplast associated genes. These molecular changes likely reflect reduced chlorophyll content, altered pigment balance, and disruption of chloroplast structure, which together modify light absorption and scattering in the visible region. This correspondence further supports the interpretation that hyperspectral signals capture integrated physiological consequence of underlying molecular stress responses.

A central outcome of this work is the demonstrated benefit of combining reflectance and fluorescence hyperspectral modalities for early drought detection. While reflectance- or fluorescence-only datasets achieved high classification accuracy at later stages of stress, they were less effective during the earliest phases when physiological changes are subtle. In contrast, models trained on the combined spectral dataset achieved substantially improved performance beginning at DAT 4–5, a window during which single-modality approaches remained limited. Across modeling frameworks, including PLS-DA, SVM, and CNN, classification accuracy, sensitivity, and specificity increased sharply during this early detection window. Although 1D-CNN models achieved high accuracy, comparable performance obtained with PLS-DA and SVM during the early detection window suggests that lightweight proxy models based on reduced spectral features can support real-time deployment. Future work will explore physics-informed neural networks (PINNs) that incorporate known spectral–physiological relationships to improve model efficiency, robustness, and generalizability under computational constraints typical of spaceflight systems. In addition, spectral band contributions to CNN predictions will be analyzed using feature attribution methods to further enhance model interpretability. Consistent with these findings, spatial classification maps further supported the biological relevance of model predictions by resolving stress-associated patterns at the plant level, enabling intuitive visualization of stress progression. Importantly, analysis of PLS-DA beta coefficients highlighted a limited set of diagnostically informative wavelengths, suggesting that compact multispectral sensor designs could be developed to capture early drought signatures in cost- and power-constrained environments such as spaceflight growth systems.

Multi-omics integration provided biological context for these phenomic patterns and revealed a coordinated, time-structured drought response in lettuce. At the molecular level, early drought responses at DAT 5 were dominated by abscisic acid (ABA)-related signaling and osmotic adjustment, including activation of guard cell and ABA-responsive transcriptional regulators (e.g., *ABF1*, *PYL4*, *PP2C56*) and accumulation of compatible solutes such as proline and raffinose-related sugars. These early responses are consistent with rapid stomatal regulation and osmotic protection that characterize initial drought perception [49,50]. As drought progressed to DAT 14, transcriptional and metabolic divergence became increasingly associated with phenylpropanoid metabolism, cell-wall remodeling, and membrane- and cuticle-related processes, including accumulation of quinate- and feruloylquinate-derived metabolites and lipid species such as sphingolipids and glycerophosphoglycerols. This temporal shift aligns with prior reports that secondary metabolism, structural reinforcement, and membrane remodeling contribute to longer-term acclimation under sustained water deficit [[51], [52], [53], [54]]. Together, these findings outline a mechanistic sequence in which early ABA-driven signaling and osmotic regulation establish the initial drought state, followed by progressive consolidation of structural and metabolic adaptations.

Although hyperspectral reflectance was not interrogated at the level of individual wavelength bands in a mechanistic sense, the molecular pathways defining the dominant drought-associated latent factor correspond closely to established spectral sensitivities in vegetation optics. Early drought-responsive processes, including ABA signaling, osmolyte accumulation, and photoprotective adjustments, influence pigment absorption and stomatal behavior that are associated with variation in the blue–green (450–560 nm), red (660–730 nm), and red-edge regions governed by chlorophyll and carotenoid dynamics [28,29,55,56]. Processes affecting mesophyll structure, turgor, and cell-wall remodeling, driven by genes such as *XET*, *EXL2*, and *ATBXL1* and metabolites including quinate and inositol derivatives, correspond to features in the near-infrared (750–1300 nm) spectral region, where reflectance is controlled primarily by internal leaf architecture [57,58]. In addition, changes in near-infrared reflectance associated with drought stress are not solely structural but are closely linked to underlying physiological and biochemical processes. Reduced water availability leads to decreased cellular turgor pressure, resulting in cell shrinkage and altered mesophyll organization, which directly influence light scattering in the NIR region [59]. Importantly, these physical changes can act as mechanical signals that trigger downstream stress-response pathways, including abscisic acid (ABA) signaling, osmotic adjustment, and transcriptional reprogramming [60]. This coupling between hydraulic status, cellular structure, and molecular signaling highlights a physio-biological mechanism through which hyperspectral features in the NIR region capture integrated plant stress responses. Later-stage membrane and cuticle modifications, reflected in lipid metabolites and cytochrome P450 activity, align with drought-associated variation in shortwave infrared regions sensitive to leaf water content and biochemical bond absorptions [31,32,61]. These correspondences provide a physiological basis for why drought discrimination was most accurate at DAT 5 in this study and establish a mechanistic bridge between multi-omics drought responses and hyperspectral signatures.

Finally, the robustness and generalizability of the hyperspectral models were validated using three independent experiments that were not used for model training. These external datasets, originally generated for transcriptomic and metabolomic profiling, showed strong agreement with spectral classification results, reinforcing the biological validity of the identified drought signatures and demonstrate the transferability of the approach across independent growouts. Together, these findings support the use of hyperspectral imaging as a biologically interpretable, non-destructive proxy for drought stress in controlled-environment agriculture and highlight its potential for integration into autonomous plant monitoring systems for terrestrial and spaceflight applications. In such systems, hyperspectral-derived stress indicators (e.g., classification probabilities) can be used to trigger irrigation control actions, such as dynamically adjusting VWC setpoints or irrigation frequency, enabling real-time, plant-responsive water management in closed-loop growth environments.

Beyond this current study, several directions are important for advancing this framework toward operational deployment in space agriculture. Future work should extend this framework to multi-stress environments relevant to spaceflight, where co-occurring factors such as water limitation, nutrient imbalance, and elevated CO_2_ interact. While hyperspectral imaging captures rich information on plant structural and energetic properties, these physical phenotypes are not fully represented within existing crop ontologies, which are primarily oriented toward morphological traits and biochemical measurements. In this study, we show that hyperspectral-derived features co-vary with underlying molecular and metabolic responses, supporting their interpretation as biologically meaningful proxies of plant physiological state. However, broader application of such approaches across controlled-environment systems requires standardized ways to describe and organize hyperspectral measurements. Variability in imaging platforms, lighting conditions, and preprocessing pipelines can limit direct comparability across studies. Establishing standardized “physical phenotyping” metadata frameworks that capture spectral features, acquisition conditions, and their biological context would facilitate reproducibility, cross-study integration, and scalable deployment of hyperspectral monitoring in controlled-environment and space-based agriculture. The two-stage phenomics-to-omics approach can be adapted to multi-label classification to resolve overlapping stress signatures. In addition, transfer to other space-relevant crops (e.g., tomato, pepper) can be achieved through species-specific calibration and retraining, leveraging conserved drought-response pathways and diagnostically informative spectral regions to enable scalable, crop-agnostic sensing systems.

## 5 Conclusion

This study demonstrates that hyperspectral imaging can serve as a biologically interpretable, non-destructive proxy for early drought stress in lettuce grown under controlled-environment conditions. Integration of reflectance and fluorescence hyperspectral data with transcriptomic and metabolomic profiling shows that drought induces coordinated optical and molecular responses that can be detected prior to visible symptoms. Classification models leveraging combined spectral modalities achieved high accuracy during early stress onset, highlighting the value of multi-modal phenomics for capturing subtle physiological perturbations.

Multi-omics analysis identified a dominant drought-associated latent factor that unified hyperspectral variation with transcriptional and metabolic reprogramming. This latent axis reflected a structured temporal response, characterized by early ABA signaling and osmotic adjustment followed by progressive engagement of phenylpropanoid metabolism, cell-wall remodeling, and membrane-associated processes. The molecular programs defining this drought state corresponded closely to established hyperspectral sensitivity regions associated with pigment dynamics, leaf structure, and water status, providing a mechanistic basis for the observed spectral discrimination.

These findings advance hyperspectral phenotyping beyond stress classification toward biological interpretability, demonstrating that optical signals encode integrated molecular states rather than isolated traits. The framework presented here supports development of compact, autonomous sensing systems capable of monitoring plant stress in real time and provides a foundation for extending hyperspectral–omics integration to additional stressors, cultivars, and crop species. In the context of space agriculture and controlled-environment food production, such biologically grounded phenotyping approaches offer a scalable pathway toward resilient, data-driven crop monitoring and management.

## Author contributions

L.M.K: Formal analysis, Data curation, Writing original draft; I.B: Conceptualization, Funding acquisition, review & editing, supervision; C.H: Data curation; M.S.K: Project administration, Funding acquisition; E.G: Formal analysis, Visualization; B.C: Investigation, review & editing; A. D: Investigation, review & editing; L.S: Investigation, review & editing; T.L: Investigation, review & editing; A. O: Conceptualization, Project administration, review & editing.

## Funding sources

This work was supported by the U.S. Department of Agriculture, Agricultural Research Service, and NASA Kennedy Space Center through Agreements No. 8042-42000-021-017N.

## Declaration of competing interest

The authors declare that they have no known competing financial interests or personal relationships that could have appeared to influence the work reported in this paper.

## Data Availability

Data collected and/or analyzed during this study can be obtained from the corresponding author upon reasonable request.
